# Mammalian tumor-like organs. 2. Mammalian adipose has many tumor features and obesity is a tumor-like process

**DOI:** 10.1186/s13027-022-00423-5

**Published:** 2022-04-08

**Authors:** A. P. Kozlov

**Affiliations:** 1 Vavilov Institute of General Genetics, Russian Academy of Sciences, 3, Gubkina Street, Moscow, Russia 117971; 2 Peter the Great St. Petersburg Polytechnic University, 29, Polytekhnicheskaya Street, St. Petersburg, Russia 195251; 3 The Biomedical Center, 8, Viborgskaya Street, St. Petersburg, Russia 194044

**Keywords:** Evolutionarily novel organs, Adipose organ, Tumor features

## Abstract

**Background:**

In previous publications, the author developed the theory of *carcino-evo-devo*, which predicts that evolutionarily novel organs should recapitulate some features of tumors in their development.

**Main text:**

Mammalian adipose is currently recognized as a multi-depot metabolic and endocrine organ consisting of several adipose tissues. Although lipid-storing cells and proteins are ancient, the adipose organ as a whole is evolutionarily novel to mammals. The adipose expansion has remarkable similarities with the growth of solid tumors. These similarities are the following: (1) The capability to unlimited expansion; (2) Reversible plasticity; (3) Induction of angiogenesis; (4) Chronic inflammation; (5) Remodeling and disfunction; (6) Systemic influence on the organism; (7) Hormone production; (8) Production of miRNAs that influence other tissues; (9) Immunosuppression; (10) DNA damage and resistance to apoptosis; (11) Destructive infiltration in other organs and tissues. These similarities include the majority of “hallmarks of cancer”. In addition, lipomas are the most frequent soft tissue tumors, and similar drugs may be used for the treatment of obesity and cancer by preventing infiltration. This raises the possibility that obesity, at least in part, may represent an oncological problem. The existing similarities between adipose and tumors suggest the possible evolutionary origin of mammalian adipose from some ancestral benign mesenchymal hereditary tumors. Indeed, using a transgenic inducible zebrafish tumor model, we described many genes, which originated in fish and were expressed in fish tumors. Their human orthologs *LEP*, *NOTCH1*, *SPRY1*, *PPARG*, *ID2*, and *CIDEA* acquired functions connected with the adipose organ. They are also involved in tumor development in humans.

**Conclusion:**

If the hypothesis of the evolutionary origin of the adipose organ from the ancestral hereditary tumor is correct, it may open new opportunities to resolve the oncological problem and the problem of the obesity epidemic. New interventions targeting *LEP*, *NOTCH1*, *SPRY1*, *PPARG*, *ID2*, and *CIDEA* gene network, in addition to what already is going on, can be designed for treatment and prevention of both obesity and tumors.

## Background

In previous publications, the author developed the theory of *carcino-evo-devo*, which describes the coevolution of normal and neoplastic development [1, 2]. I formulated the hypothesis of evolution by tumor neofunctionalization (below I will call it "the main hypothesis"), which suggested that the evolutionary role of hereditary tumors might consist in supplying evolving multicellular organisms with extra cells masses for expression of evolutionarily novel genes and the origin of new cell types, tissues, and organs [1, 2].

Several non-trivial predictions of the main hypothesis have been confirmed in my laboratory ([1–4], reviewed in [5]). One non-trivial prediction of the main hypothesis is that evolutionarily novel organs if they indeed originated from hereditary tumors or tumor-like structures, should recapitulate some features of tumors in their development. That is why in previous articles [2, 3, 5] I was looking for the data that might confirm this prediction in the literature, and also performed some experiments in my lab. The first paper in this series [5] reviewed the evidence that evolutionarily novel organs such as the placenta, mammary gland, prostate, and infantile brain indeed have many features of tumors including the regulated invasiveness at certain stages of their development and higher incidence of cancer. In that paper, I suggested calling evolutionarily new normal organs, which have many tumor features, the tumor-like organs for brevity [5].

In the present paper, the author reviews the evidence that mammalian adipose, the other evolutionarily novel organ of mammals, has many tumor features as well, and obesity is a tumor-like process. A hypothesis of the possible evolutionary origin of mammalian adipose from hereditary mesenchymal tumors is formulated and supporting data obtained in the author’s lab are discussed.

## Main text

### Mammalian adipose is a metabolic and endocrine organ evolutionarily novel to mammals

Adipose is a metabolic and endocrine organ operating “as a structured whole” [6, 7]. The concept of adipose as a large multi-depot organ with discrete anatomy was developed by S. Cinti [6, 8–10]. The adipose organ consists of white adipose tissue and brown adipose tissue distributed in a series of subcutaneous and visceral depots. Each depot of the organ has its own vascular and nerve supply. White adipose tissue (WAT) is involved in triglyceride/energy storage, and brown adipose tissue (BAT)—in energy expenditure.WAT and BAT differ in morphology and location [10]. The third type of fat, beige adipose tissue, resembles brown adipose morphologically and functionally, but its development is closer to the development of white adipose tissue [10, 11]. Some authors also consider bone marrow adipose as a separate type of adipose tissue [12].

Adipose has a mesodermal origin, but white and brown adipocytes develop from separate precursor cells, through separate differentiating lineages, and by using different differentiation factors. Beige adipocytes develop from precursors of white adipocytes [13, 14]. Adipose cells can also originate from tumor cells—trans-differentiation of breast cancer cells into functional adipocytes was reported [15].

Although the storage of energy in lipids is evolutionarily conserved, and lipid-storing cells and proteins (FIT) are ancient [16, 17], the adipose organ is evolutionarily novel to mammals [18]. BAT has not been described in fishes, amphibians, reptiles, or birds, and is present only in higher mammals [19, 20]. The overview of the evolution of adipose tissue depots shows the gradual accumulation of features such as the way of fat storage, leptin, BAT, uncoupling protein-1 (UCP-1) in BAT, and thermoregulation in mammalian evolutionary lineage [19]. Adipose organ acquired several fundamental metabolic functions since the early evolution of mammals as an adaptation to new diets and thermoregulation [18]. Adipose plays a central role in the energy metabolism and maintenance of glucose homeostasis [21]. Adipose associated with other organs has a diversity of additional functions and adaptations, and participate in morphogenetic processes [17].

### Similarities of mammalian adipose to tumors

#### The capability of unlimited expansion

The main similarity of adipose to tumors is its capability to almost unlimited expansion. Variations in nutrition or environmental temperature cause dramatic anatomical changes in the adipose organ. In obesity, it can increase its mass tremendously. Adipose tissue expands due to hypertrophy and hyperplasia of adipocytes. The authors stress that there are **“**remarkable similarities between adipose expansion and growth of solid tumors” [22]. The adipose expansion may lead to a pathological condition, i.e. obesity and related metabolic disorders. Obesity is a risk factor for developing lipomas and other types of tumors, not only adipose tumors.

#### Relatively high prevalence of mammalian adipose tumors

Adipose tumors comprise a large group of human tumors. Lipomas are the most frequent soft tissue tumors (50% of all soft-tissue masses) and are found in 2% of the population [23–25]. The mammary gland and prostate, the other evolutionarily novel mammalian organs, are also characterized by the highest incidence of tumors [26]. Lipomas are benign tumors, while liposarcomas are malignant adipose tumors with different degrees of malignancy. Liposarcomas are the most prevalent soft tissue malignancy [24, 27–29].

#### The remarkable plasticity of mammalian adipose

The reversible plasticity of cancer cells is well known (reviewed in [2]). Cell plasticity is defined as “the ability of cells to change their phenotypes without genetic mutations in response to environmental cues” [30]. Neoplasms have been associated with increased plasticity, although cell plasticity was first observed during normal development.

The plasticity of the adipose is remarkable and reminds that of tumors. The adipose organ can increase in size or regress, depending on the energy balance. Earlier studies suggested adipocytes transdifferentiation during cold exposure, physical exercise, lactation, and obesity [9, 10]. At present, the appearance of beige adipocytes in WAT depots after cold exposure or stimulation (beiging or browning of WAT) is viewed as direct transdifferentiation of white adipocytes, or as differentiation from progenitor cells. The process of WAT beiging is reversible [31–33].

Adipose is involved in the development of the mammary gland, the other novel mammalian organ with many tumor features. Subcutaneous adipose depots participate in mammary gland formation during lactation and pregnancy [9]. Earlier data suggested that mammary adipocytes transdifferentiate into mammary epithelial cells during mammary gland development [8, 9]. The latest cell lineage tracing studies showed that white adipocytes in the mammary gland and skin can reversibly dedifferentiate into preadipocytes [33], and adipocyte progenitor cells can differentiate into epithelial cells of the mammary gland [34]. White adipocytes can reversibly transdifferentiate into myofibroblasts and cancer-associated fibroblasts during fibrosis and cancer, and dedifferentiate in liposarcomas [33]. These new data support the earlier evidence on the possibility of mammary adipocytes transdifferentiation into mammary epithelial cells [8, 9]. On the other hand, as already mentioned above, EMT-derived breast cancer cells can trans-differentiate into post-mitotic functional adipocytes [15]. Mammary adipose controls breast cancer progression: mammary preadipocytes act locally by releasing cytokines, growth factors, and extracellular matrix components [35]. Interestingly, liposarcomas are very rare in the mammary gland that is characterized by the high incidence of cancer [27, 28].

#### Adipose expansion induces angiogenesis

Tumor growth induces angiogenesis. Similarly, adipose expansion also induces angiogenesis. The angiogenic activity of adipocytes is connected with the secretion of pro-angiogenic molecules. The newly formed vasculature is important for adipogenesis. Angiogenesis is a rate-limiting step for adipose expansion [22, 36].

#### Chronic inflammation

Like solid tumors, adipose expansion is connected with hypoxia. Hypoxia is one of the factors that cause macrophage infiltration of obese adipose tissue. The other factors are adipocyte death, chemotactic regulation, and fatty acid flux [22]. Infiltrated macrophages participate in adipose tissue inflammation. Saturated fatty acids released from adipocytes are ligands for Toll-like receptor 4 complex located on macrophages. Their interaction induces inflammatory changes in macrophages, which include TNFα production. A paracrine loop involving saturated fatty acids and TNFα causes chronic inflammatory responses in adipose tissue [37]. Low-grade chronic inflammation connected with obesity is a risk factor for many cancers [38].

Similar interactions between endogenous ligands and pathogen sensors occur in tumors which are also chronic inflammatory diseases. Tumor-associated macrophages are a major type of inflammatory cells infiltrating most tumors. The recruitment of immune cells and increased expression of inflammatory mediators in tumors constitute the phenomenon of tumor-elicited inflammation. Inflammation is connected with the initiation of tumors and with different stages of tumor progression [38].

#### Remodeling and disfunction

Considerable changes in obese adipose tissue, including changes of extracellular matrix (ECM), adipogenesis, and metabolism, constitute adipose tissue remodeling [22, 37]. Tumors also undergo considerable dynamic changes and remodeling of chromatin [39], ECM [40, 41], vasculature [42], and metabolism [43].

Inflammation, fibrosis, and impaired angiogenesis cause disfunction of adipose organ, which leads to obesity and related metabolic complications [44, 45]. The loss of function and differentiation features is also connected with tumor development (reviewed in [2]).

#### DNA damage and resistance to apoptosis

Obesity-related inflammation and oxidative stress cause DNA damage in adipocytes and other tissues [46] that can lead to obesity-related carcinogenesis [47]. This suggests the similarity of DNA damage mechanisms in obesity and carcinogenesis. There are even more similarities. DNA damage in obese adipocytes activates the p53 pathway [48], as it does in tumors [49]. p53 negatively regulates both tumorigenesis and adipogenesis [50].

DNA damage is an initial stage of molecular processes that leads to genomic instability. Genomic instability is a feature of most tumors [51].

In hereditary cancers, mutations in DNA repair genes cause genomic instability [51]. CIDE proteins involved in regulating lipid metabolism belong to the family of Cell death-Inducing DNA fragmentation Factor Alpha (DFFA)-like Effector proteins. They may participate in the DNA fragmentation step in apoptosis [52, 53].

Apoptosis of adipocytes is anticipated in the stressful obese environment. However, anti-apoptotic factors such as YAP, TAZ, and Bcl2 are activated in obese adipocytes protecting them from cell death [54], a situation similar to that in tumor cells. Survivin, another potent apoptosis inhibitor, is upregulated in obesity by inflammation and oxidative stress. Survivin is also the regulator of lipid storage and metabolism. On the other hand, survivin is an oncogene expressed in most tumors. Thus, survivin is the direct molecular link between obese adipose and tumors [55].

#### Systemic influence in the organism

Adipose, as the central energy metabolism regulator, influences other tissues' metabolism. Adipose regulates the other tissues' metabolism according to the nutritional balance of the organism. Obesity causes systemic metabolic disorders such as insulin resistance and diabetes [7, 56, 57]. The systemic influence of tumors on the organism has also been known since long ago [58, 59].

Tumor cells have a higher rate of glucose consumption than normal cells [60–62]. That is why tumors are called "the glucose trap" [61]. Adipose has enhanced glucose utilization during accelerated body-fat recovery (catch-up fat), which is connected with muscle-adipose glucose redistribution [63, 64]. The authors use the terminology “the glucose sink” to describe the role of enhanced de novo lipogenesis in regulating glycemia during catch-up growth [65].

Cancer can cause cachexia, a wasting syndrome. Cachexia is associated with systemic inflammation connected with tumors and tumor-induced changes in the metabolism [66, 67]. Brown adipose tissue and energy expenditure are increased in cachexic patients. The uncoupling protein UCP3, found in brown adipose tissue, is increased in cancer and is connected with high energy expenditure [68]. Tumor-derived parathyroid hormone-related protein (PTHrP) is involved in adipose beiging, energy-wasting, and cancer cachexia [69].

##### Hormone production

Both adipose and tumors produce hormones. The concept of adipose as an endocrine organ is widely accepted [7, 56, 70, 71]. Adipose organ secretes several hormones (adipokines and batokines [72]) and classical cytokines, especially TNFα. The energy metabolism is regulated by adipokines leptin, adiponectin, resistin, and others [56]. Leptin has an important physiological role in the central control of energy and lipid metabolism and the regulation of metabolism-immune system interplay (immunometabolism) [73]. Mammalian leptin is defined as a lipostatic signal, which regulates energy balance by controlling food intake. It also regulates glucose homeostasis maintenance and participates in the regulation of immunometabolism [56, 73].

The phenomenon of hormone secretion by non-endocrine tumors is known as “ectopic” hormone production [74–76]. It causes unique clinical syndromes or endocrine paraneoplastic syndromes. These syndromes represent an important cause of morbidity and mortality [77]. Ectopic hormones are similar to normal hormones, but in tumors, they usually are present in lower amounts per unit mass than in normal endocrine organs.

##### miRNA production

Both adipose and tumors produce miRNAs that influence other tissues. For example, adipose-derived circulating miRNAs can regulate gene expression in other tissues [78]. Tumor-derived immune-modulatory miRNAs influence cancer immune surveillance and immune escape [79].

##### Immunosuppression

Obesity and related metabolic syndrome cause negative effects on immunity [56, 73, 80, 81]. Cancer immunosuppression is also a well-known phenomenon [82–85]. Obese metabolism suppresses antitumor immunity [86].

#### Destructive infiltration in other organs and tissues

Ectopic lipid deposition (ELD) in skeletal muscles, heart, liver, pancreas, placenta, and kidney during obesity is a major cause of metabolism distortion [87–89]. ELD is caused by the formation of lipid droplets in the organ's parenchymal cells; in adipocytes originated by differentiation of resident adipogenic progenitors; or in adipocytes differentiated after infiltration of organs with adipocyte progenitors from subcutaneous adipose tissue [90–93]. Infiltration of adipocyte progenitors in other organs with a negative influence on these organs' functions is the most important similarity with tumor metastasis.

CXCL12/CXCR4 chemokine axis participates in tumor progression and metastasis [94–96]. Adipocyte progenitors trafficking is also regulated by the CXCL12/CXCR4 axis [93].

#### Similar drugs may be used for the treatment of obesity and cancer

Thiazolidinediones (or glitazones) are used for the treatment of type 2 diabetes. By binding peroxisome proliferator-activated receptor gamma (PPARγ) they promote the maturation of adipocytes. They also suppress tumor cell invasion, migration, and invasiveness through CXCL12/CXCR4 pathway. In addition, it was found that treatment of mice with pioglitazone (a member of the glitazone group) prevents infiltration of adipocyte progenitors in skeletal muscles [93]. The other class of antidiabetic drugs—biguanides—also act as anti-carcinogens and inhibitors of tumor growth [97–99].

#### The connection between obesity and cancer

Obesity-related inflammation and oxidative stress cause DNA damage that can lead to obesity-related carcinogenesis [47].

Obesity and type 2 diabetes are associated with the risk of cancer and cancer-related mortality, as shown in epidemiological studies [100]. The link can be related to the insulin/insulin-like growth factor (IGF) system [57, 100]. Throughout evolution, this system “has integrated the control of tissue growth with metabolic status" [57]. Tumors are connected with the insulin/IGF system and systemic metabolism. The development and progression of several types of cancer are determined by the insulin/IGF system [98]. The factors that play a role in this connection include insulin resistance, hyperinsulinemia, increased levels of insulin growth factors (IGFs), hormones, and inflammatory markers [57, 100, 101].

Using Paget’s terminology of “seed” and “soil” as related to tumor metastasis [102], Holly and co-authors describe the internal milieu of obese individuals, or "soil," as containing high levels of glucose, insulin, insulin-like growth factors, inflammatory cytokines, and adipokines. These authors believe that such an environment stimulates the latent neoplastic lesions, the "seeds," to progress to clinical cancer [57]. Indeed, obese metabolism suppresses antitumor immunity [86].

The paradox of some benefits of obesity in cancer is also known: moderate overweight and early obese states can improve the survival and response to therapy [103, 104].

#### Mammalian adipose is a tumor-like organ, and obesity is a tumor-like process

Thus, adipose, an evolutionarily young organ of mammals, shares many features with tumors. Adipose unlimited expansion is similar to tumor growth, and lipomas are the most frequent soft tissue tumors. Chronic inflammation is characteristic of obese adipose and tumors. Both adipose and tumors exert systemic metabolic and immunological influence on the organism; both participate in paracrine and endocrine interactions with other tissues; both produce miRNAs that influence other tissues; both are characterized by plasticity, induce angiogenesis and participate in morphogenetic processes. Tumors act as “glucose trap”, and adipose during catch-up fat—as “glucose sink”. Obese adipose and tumors can cause immunosuppression; obese adipose and tumors are connected with remodeling and disfunction, with DNA damage and cell death resistance. Most important, adipose cells can metastasize into normal organs and impair their functions, similarly to malignant tumors. Finally, the same drugs and interventions are used against obesity, diabetes, ectopic lipid deposition, and tumors. Many of the common features of tumors and adipose organ are in the list of so-called "hallmarks of cancer" [105], and many of them are connected with the obese state of the adipose organ.

As discussed earlier, other evolutionarily novel organs of mammals, such as the placenta, mammary gland, and prostate, also have many tumor features [2, 5]. However, evolutionarily older organs are characterized by lower cancer rates [26] and do not have (or have fewer) tumor features. The author suggested calling normal organs, which have many tumor features, the "tumor-like organs" [3, 5]. We may conclude that mammalian adipose is a tumor-like organ and obesity is a tumor-like process.

### The possible origin of mammalian adipose from ancestral mesenchymal hereditary tumors

The mammalian adipose organ's tumor features suggest its recent evolutionary origin from ancestral hereditary tumors. Following the main hypothesis, the adipose organ's origin may be represented as follows. Some diffuse mesenchymal hereditary tumors in eutherian ancestors, which produced several biologically active compounds (future adipokine hormones), acquired the capability to synthesize and accumulate fat using pre-existing and evolutionarily novel genes. Accumulation of lipids inhibited the potential of progression to malignancy ("gain fat—loose metastasis," [15]) and, together with future adipokine substances, was selected in ancestral Mammalia for control of energy metabolism in connection to the nutritional status of the organism, as an adaptation to new diets and thermoregulation. As a result of this evolutionary process, the evolutionarily novel mammalian organ involved in the storage and expenditure of energy with many ancestral tumor features—the adipose organ—originated.

The lab of the author has already obtained the evidence in support of a hypothesis specific to the origin of mammalian adipose organ.

In our previous article [4], we studied fish genes expressed in transgenic zebrafish inducible tumors, tumors after regression, and spontaneous zebrafish tumors. Among these genes, using the Orthologous Matrix (OMA) approach, we selected genes evolutionarily novel to fishes (as compared to lamprey, myxine, and other organisms in fish evolutionary lineage), and studied their human orthologs. We described many human orthologs that acquired progressive functions (such as involvement in the development of the placenta, mammary gland, lungs, neocortex, according to Gene Ontology studies), which are not encountered in fish [4]. Several of those human genes with progressive functions—*LEP*, *NOTCH1, SPRY1*, *PPARG, ID2*, and *CIDEA* genes—also acquired functions connected with the adipose organ.

Thus, human *LEP*, which encodes leptin, became the central regulator of energy metabolism in mammals. It is involved in beige/brown fat cell differentiation regulation [4, 106] and lipostatic function (fish leptin is not an adipostat [107, 108]). Mammalian leptin is also involved in thermoregulation [109, 110].

*NOTCH1* regulates adipose browning, energy metabolism, and thermogenesis [111, 112].

*SPRY1* is mandatory for the initiation and regulation of adipogenesis, for maintaining proliferation and differentiation of human adipose stem/progenitor cells (ASCs). It is induced in ASCs after weight loss [113, 114]. *SPRY1* can suppress *PPARG* [115].

*PPARG*, the ortholog of the fish *pparg* gene, was selected in [4] because of its involvement in placenta development. Peroxisome proliferator-activated receptor gamma (PPARγ), encoded by *PPARG*, is the target of thiazolidinediones antidiabetic treatment, as discussed above. PPARγ participates in the differentiation of adipocytes and activation of thermogenic gene expression in brown adipocytes [116]. PPARγ is a major regulator of adipocyte differentiation and function [117]. It plays a role in lipodystrophy, obesity, and diabetes [118] and can downregulate *LEP* gene expression [104, 119].

*ID2* stimulates PPARγ expression, adipocyte differentiation, and adipogenesis. Its expression is elevated in adipose tissues during obesity [120].

The *CIDEA* gene was also found among human orthologs of novel fish genes expressed in fish tumors [4]. CIDE proteins are associated with lipid droplets and regulate lipid metabolism. CIDE protein family includes CIDEA, CIDEB, and CIDEC proteins [52, 53]. In mice, CIDEA is a marker of brown and brite adipocytes [121]. In humans, the *CIDEA* gene regulates adipocyte beiging [122]. It means that in mammals *CIDEA* gene also acquired progressive functions not encountered in fish. Transcription of *CIDEA* gene is activated by PPARγ [52].

Each of the *LEP*, *NOTCH1*, *SPRY1*, *PPARG*, *ID2*, and *CIDEA* genes is also involved in tumor development in humans.

Leptin is overexpressed in breast cancer [104] and many other types of cancer [123], has a role at different levels and participates in cancer progression. Its activation results in the activation of multiple oncogenic pathways. Leptin oncogenic functions are reinforced through crosstalk with oncogenes, e.g. *NOTCH* [104].

*NOTCH1* has both oncogenic and tumor suppressor abilities [124, 125].

*SPRY1* is downregulated in some tumors and overexpressed in other tumors. Depending on the cellular context, it may serve either as a tumor suppressor or tumor promoter. *SPRY1* expression is essential for induction, maintenance, and progression of tumors [126–131].

PPARγ plays oncogenic and tumor suppressor roles. PPARγ functions as a tumor suppressor in colon, lung, pancreatic, and breast cancers. A tumor-promoting role for PPARγ has been suggested in a variety of cancers as well [117].

ID family of proteins participates in the regulation of pathways essential to the progression of cancer. *ID* gene transcription is sensitive to signals from the cellular environment including oncoproteins. Depending on the context, ID proteins can play tumor-promoting or tumor-suppressing roles [132–134]. The tumor-suppressive role of *ID2* has been described in [135, 136], and its oncogenic role—in [137].

CIDE proteins control lipid droplets' size and metabolism [53]. Lipid droplets actively participate in tumor processes and accumulate in a variety of cancer cells [138, 139]. *CIDEA* controls the beiging of adipocytes [122], and beige adipocytes contribute to breast cancer progression [140]. *CIDEA* plays an important role in human cachexia [141]. CIDE proteins were originally discovered as apoptotic proteins. They induce caspase-independent cell death in various cell types [53] that can be connected with cancer processes.

Once more, orthologs of *LEP*, *NOTCH1*, *SPRY1*, *PPARG*, *ID2,* and *CIDEA* genes originated in fishes and were expressed in fish tumors. In humans, these genes acquired progressive functions not encountered in fishes, including functions connected with mammalian adipose organ, form a gene network with mutual influences, and participate in tumor processes. These genomic and transcriptomic data support the possibility of mammalian adipose origin from ancestral hereditary tumors and the tumor-like nature of mammalian adipose.

The hypothesis of adipose origin by ancestral tumor neofunctionalization is also strongly supported by the experimental cytology approach: it was demonstrated that breast cancer cells can trans-differentiate, in experimental conditions of adipogenesis, into post-mitotic functional adipocytes with the loss of malignancy [15]. Similarly, by trans-differentiation after the expression of evolutionarily novel genes and gene combinations involved in adipogenesis, mammalian adipocytes could originate in evolution from ancestral hereditary tumor cells.

The possible origin of mammalian adipose from ancestral tumors is in correspondence with other examples of hereditary tumors, which played roles in the origin of new cell types, tissues, and organs discussed in our previous publications [1–3, 5].

## Conclusion

The ongoing synthesis of evolutionary biology and health sciences attempts to find evolutionary roots of disease [1, 142, 143]. If the hypothesis of the evolutionary origin of the adipose organ from the ancestral hereditary tumor is correct, it may help find new clues to obesity and cancer. Approaches developed to prevent and treat obesity may be examined to prevent and treat tumors and vice-versa. Empirically, some of such approaches are already underway [144]. Cancer metabolism is currently being studied for therapeutic opportunities, along with calorie restriction interventions for the prevention and treatment of cancer. Obese adipose supports tumor growth in various ways, and interventions aimed at metabolic disorders caused by adipose expansion may also be effective against tumors.

Our hypothesis may add a theoretical ground to such studies and may open new opportunities to resolve the oncological problem and the problem of the obesity epidemic. New interventions targeting *LEP*, *NOTCH1, SPRY1*, *PPARG*, *ID2*, and *CIDEA* gene network, in addition to what already is going on, can be designed for treatment and prevention of both obesity and tumors.

## Data Availability

Not applicable.

## Abbreviations

ASCs: Adipose stem/progenitor cells
BAT: Brown adipose tissue
DFFA: DNA fragmentation factor alpha
ECM: Extracellular matrix
ELD: Ectopic lipid deposition
EMT: Epithelial-mesenchymal transition
FIT: Fat storage-inducing transmembrane
IGF: Insulin-like growth factor
OMA: Orthologous matrix
PPARγ: Peroxisome proliferator-activated receptor gamma
PTHrP: Tumor-derived parathyroid hormone-related protein
UCP: Uncoupling protein
WAT: White adipose tissue
